# 1-(2,6-Difluoro­benzo­yl)-3-(2,3,5-tri­chloro­phen­yl)urea

**DOI:** 10.1107/S1600536808032029

**Published:** 2008-10-11

**Authors:** Sheng-Jiao Yan, Chao Huang, Yan-Mei Li, Yu-Yun Yan, Jun Lin

**Affiliations:** a School of Chemical Science and Technology, Key Laboratory of Medicinal Chemistry for Natural Resources, (Ministry of Education), Yunnan University, Kunming 650091, People’s Republic of China

## Abstract

The asymmetric unit of the title compound, C_14_H_7_Cl_3_F_2_N_2_O_2_, contains two unique molecules. The 2,3,5-trichloro­phenyl ring is almost coplanar with the urea group in both molecules, whereas the 2,6-difluoro­phenyl ring is twisted from the urea plane by 54.83 (10)° in one molecule and 60.58 (10)° in the other. An intra­molecular N—H—O hydrogen bond stabilizes the mol­ecular conformation. The crystal packing is formed by inter­molecular N—H—O hydrogen bonds and F⋯F inter­actions [2.841 (2) Å].

## Related literature

For general background, see: Yan *et al.* (2003 ▶). For synthetic details, see: Lin *et al.* (2003 ▶, 2005 ▶). 
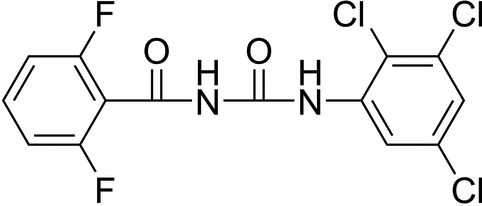


## Experimental

### 

#### Crystal data

C_14_H_7_Cl_3_F_2_N_2_O_2_
*M* *_r_* = 379.57
Monoclinic, 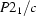

*a* = 7.1669 (4) Å
*b* = 22.8228 (12) Å
*c* = 18.2885 (10) Å
β = 94.768 (2)°
*V* = 2981.1 (3) Å^3^
*Z* = 8
Mo *K*α radiation
μ = 0.65 mm^−1^
*T* = 113 (2) K
0.24 × 0.14 × 0.12 mm

#### Data collection

Rigaku Saturn diffractometer
Absorption correction: multi-scan (*CrystalClear*; Rigaku, 2006 ▶) *T*_min_ = 0.860, *T*_max_ = 0.927
27779 measured reflections
7091 independent reflections
6011 reflections with *I* > 2σ(*I*)
*R*_int_ = 0.050

#### Refinement

*R*[*F* ^2^ > 2σ(*F* ^2^)] = 0.039
*wR*(*F* ^2^) = 0.089
*S* = 1.07
7091 reflections
431 parameters
H atoms treated by a mixture of independent and constrained refinement
Δρ_max_ = 0.29 e Å^−3^
Δρ_min_ = −0.29 e Å^−3^

### 

Data collection: *CrystalClear* (Rigaku, 2006 ▶); cell refinement: *CrystalClear*; data reduction: *CrystalClear*; program(s) used to solve structure: *SHELXS97* (Sheldrick, 2008 ▶); program(s) used to refine structure: *SHELXL97* (Sheldrick, 2008 ▶); molecular graphics: *SHELXTL* (Sheldrick, 2008 ▶); software used to prepare material for publication: *CrystalStructure* (Rigaku, 2006 ▶).

## Supplementary Material

Crystal structure: contains datablocks global, I. DOI: 10.1107/S1600536808032029/jh2065sup1.cif

Structure factors: contains datablocks I. DOI: 10.1107/S1600536808032029/jh2065Isup2.hkl

Additional supplementary materials:  crystallographic information; 3D view; checkCIF report

## Figures and Tables

**Table 1:** Hydrogen-bond geometry (Å, °)

*D*—H⋯*A*	*D*—H	H⋯*A*	*D*⋯*A*	*D*—H⋯*A*
N1—H1⋯Cl1	0.89 (2)	2.46 (2)	2.9126 (15)	111.8 (16)
N1—H1⋯O2	0.89 (2)	1.88 (2)	2.641 (2)	141.5 (19)
N2—H2⋯O1^i^	0.82 (2)	2.00 (2)	2.8205 (19)	173 (2)
N3—H3⋯Cl4	0.80 (2)	2.43 (2)	2.8944 (16)	118.2 (19)
N3—H3⋯O4	0.80 (2)	1.99 (2)	2.658 (2)	140 (2)
N4—H4⋯O3^ii^	0.91 (2)	1.93 (2)	2.8378 (18)	176 (2)

Symmetry codes: (i) 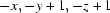; (ii) 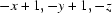.

**Fig. 1.**
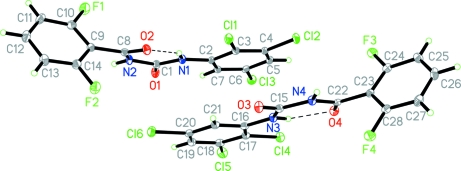
View of (I) showing 30% displacement ellipsoids (arbitrary spheres for the H atoms).

**Fig. 2.**
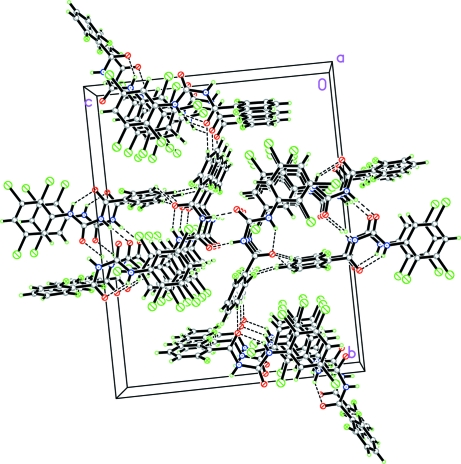
The crystal packing of complex 1 showing the hydrogen bonds as broken lines. Symmetry code: (i) -x, -y + 1, -z + 1; (ii) -x + 1, -y + 1, -z.

**Fig. 3.**
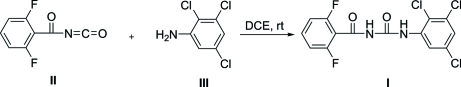
The formation of the title compound.

## supplementary crystallographic information

### Comment

Derivatives of benzoylphenylureas (BPUs) are kind of insect growth regulators (IGRs), interferes the chitin synthesis in target pests, causing death or abortive development. BPUs posses high selectivity, low acute toxicity for mammals. At the time, the different groups on the phenyl that have different bioactivity. So research the configuration of the different compound is important to find more potent insecticide. The title compound (I) (Fig. 1), C_14_H_7_Cl_3_F_2_N_2_O_2_, which possesses high bioactivity to pests (Yan *et al.*, 2003).

The geometrical parameters for (I) (Table 1) show the conjugation present: the length of the C1═O1 and C═O2 double bond is greater than that of a normal C═O double bond. The lengths of the C1—N1, C1—N2, C8—N2 bonds are shorter than that of normal C—N single bonds. The 2,3,5-trichlorophenyl ring of the title compound is almost coplanar with the urea group, whereas the 2,6-difluorophenyl ring is twisted from the urea plane by 60.58 (10)°. An intramolecular N—H—O hydrogen bond stabilizes the molecular conformation. The crystal packing of the title compound formed by intermolecular N—H—O hydrogen bonds and F···F bond (Fig 2).

### Experimental

A solution of 2,6-difluorobenzoyl isocyanate (II) (10 mmol, 1.0 equiv.) in 1,2-dichloroethane (10 ml) was added to a stirred solution of 2,3,5-trichloroaniline (III) (10 mmol, 1.0 equiv.) in dry 1,2-dichloroethane (20 ml) and stirred at room temperature for 24 hrs, the solvent was removed *in vacuo* and the residue was recrystallized with ethyl acetate to give desired compounds as white needle-crystals (I) in 93% yield (Lin *et al.*, 2003; Lin *et al.*, 2005). The desire product recrystallized from acetone (m.p. 517 K).

### Refinement

In the absence of significant anomalous dispersion effects, Freidel pairs were merged; the absolute configuration was assigned on the basis of the known configuration of the starting material. All H atoms were placed in idealized positions and refined with riding constraints, with C—H distances in the range 0.93–0.96 Å and with *U*_iso_(H) = 1.2 or 1.5 times *U*_eq_(C).

### Crystal data

**Table:**

C_14_H_7_Cl_3_F_2_N_2_O_2_	*D*_x_ = 1.691 Mg m^−^^3^
*M**_r_* = 379.57	Melting point: 517 K
Monoclinic, *P*2_1_/*c*	Mo *K*α radiation, λ = 0.71070 Å
*a* = 7.1669 (4) Å	Cell parameters from 6247 reflections
*b* = 22.8228 (12) Å	θ = 1.8–27.9°
*c* = 18.2885 (10) Å	µ = 0.65 mm^−^^1^
β = 94.768 (2)°	*T* = 113 K
*V* = 2981.1 (3) Å^3^	Prism, colourless
*Z* = 8	0.24 × 0.14 × 0.12 mm
*F*(000) = 1520	

### Data collection

**Table:**

Rigaku Saturn diffractometer	7091 independent reflections
Radiation source: rotating anode	6011 reflections with *I* > 2σ(*I*)
confocal	*R*_int_ = 0.050
Detector resolution: 7.31 pixels mm^-1^	θ_max_ = 27.9°, θ_min_ = 1.8°
ω and φ scans	*h* = −9→9
Absorption correction: multi-scan (CrystalClear; Rigaku, 2006)	*k* = −30→30
*T*_min_ = 0.860, *T*_max_ = 0.927	*l* = −24→24
27779 measured reflections	

### Refinement

**Table:**

Refinement on *F*^2^	Primary atom site location: structure-invariant direct methods
Least-squares matrix: full	Secondary atom site location: difference Fourier map
*R*[*F*^2^ > 2σ(*F*^2^)] = 0.039	Hydrogen site location: inferred from neighbouring sites
*wR*(*F*^2^) = 0.089	H atoms treated by a mixture of independent and constrained refinement
*S* = 1.07	*w* = 1/[σ^2^(*F*_o_^2^) + (0.0419*P*)^2^ + 0.1727*P*] where *P* = (*F*_o_^2^ + 2*F*_c_^2^)/3
7091 reflections	(Δ/σ)_max_ = 0.001
431 parameters	Δρ_max_ = 0.29 e Å^−^^3^
0 restraints	Δρ_min_ = −0.29 e Å^−^^3^

### Special details

**Table:**

Geometry. All e.s.d.'s (except the e.s.d. in the dihedral angle between two l.s. planes) are estimated using the full covariance matrix. The cell e.s.d.'s are taken into account individually in the estimation of e.s.d.'s in distances, angles and torsion angles; correlations between e.s.d.'s in cell parameters are only used when they are defined by crystal symmetry. An approximate (isotropic) treatment of cell e.s.d.'s is used for estimating e.s.d.'s involving l.s. planes.
Refinement. Refinement of *F*^2^ against ALL reflections. The weighted *R*-factor *wR* and goodness of fit *S* are based on *F*^2^, conventional *R*-factors *R* are based on *F*, with *F* set to zero for negative *F*^2^. The threshold expression of *F*^2^ > σ(*F*^2^) is used only for calculating *R*-factors(gt) *etc*. and is not relevant to the choice of reflections for refinement. *R*-factors based on *F*^2^ are statistically about twice as large as those based on *F*, and *R*- factors based on ALL data will be even larger.

### Fractional atomic coordinates and isotropic or equivalent isotropic displacement parameters (Å^2^)

**Table:**

	*x*	*y*	*z*	*U*_iso_*/*U*_eq_	
Cl1	0.03364 (7)	0.50654 (2)	0.15961 (2)	0.02733 (12)	
Cl2	−0.07937 (7)	0.40202 (2)	0.05408 (2)	0.03038 (12)	
Cl3	−0.17311 (7)	0.26475 (2)	0.28607 (3)	0.03149 (12)	
Cl4	0.34943 (7)	0.283841 (19)	0.19434 (2)	0.02671 (11)	
Cl5	0.36147 (7)	0.28306 (2)	0.36548 (2)	0.03282 (13)	
Cl6	0.54168 (7)	0.51085 (2)	0.37218 (2)	0.02918 (12)	
F1	−0.17544 (16)	0.64246 (6)	0.45899 (7)	0.0423 (3)	
F2	0.44718 (15)	0.65048 (6)	0.39926 (7)	0.0414 (3)	
F3	0.04346 (14)	0.34365 (5)	−0.10568 (6)	0.0303 (3)	
F4	0.67828 (15)	0.39222 (5)	−0.10684 (6)	0.0327 (3)	
O1	−0.02709 (19)	0.44667 (6)	0.42993 (6)	0.0291 (3)	
O2	0.14058 (17)	0.59069 (6)	0.31730 (6)	0.0248 (3)	
O3	0.51390 (18)	0.48843 (5)	0.09749 (6)	0.0247 (3)	
O4	0.33939 (17)	0.32720 (5)	0.01436 (6)	0.0240 (3)	
N1	0.0186 (2)	0.48145 (7)	0.31516 (8)	0.0214 (3)	
N2	0.0531 (2)	0.54179 (7)	0.41771 (8)	0.0225 (3)	
N3	0.4215 (2)	0.39641 (7)	0.12945 (8)	0.0202 (3)	
N4	0.4259 (2)	0.42336 (7)	0.00736 (8)	0.0201 (3)	
C1	0.0123 (3)	0.48592 (8)	0.38878 (9)	0.0221 (4)	
C2	−0.0276 (2)	0.43225 (8)	0.27132 (9)	0.0197 (4)	
C3	−0.0277 (2)	0.43974 (8)	0.19488 (9)	0.0212 (4)	
C4	−0.0753 (2)	0.39311 (8)	0.14825 (9)	0.0225 (4)	
C5	−0.1203 (2)	0.33913 (8)	0.17552 (10)	0.0246 (4)	
H5	−0.1524	0.3073	0.1434	0.029*	
C6	−0.1176 (2)	0.33237 (8)	0.25079 (10)	0.0226 (4)	
C7	−0.0722 (2)	0.37795 (8)	0.29909 (9)	0.0215 (4)	
H7	−0.0717	0.3721	0.3505	0.026*	
C8	0.1103 (2)	0.59011 (8)	0.38219 (9)	0.0205 (4)	
C9	0.1360 (2)	0.64407 (8)	0.42805 (9)	0.0207 (4)	
C10	−0.0058 (3)	0.66965 (9)	0.46427 (10)	0.0270 (4)	
C11	0.0158 (3)	0.72142 (9)	0.50213 (11)	0.0331 (5)	
H11	−0.0859	0.7382	0.5250	0.040*	
C12	0.1879 (3)	0.74862 (9)	0.50626 (10)	0.0314 (5)	
H12	0.2058	0.7840	0.5334	0.038*	
C13	0.3350 (3)	0.72521 (8)	0.47147 (10)	0.0291 (4)	
H13	0.4537	0.7440	0.4742	0.035*	
C14	0.3047 (3)	0.67426 (8)	0.43304 (10)	0.0252 (4)	
C15	0.4574 (2)	0.43923 (8)	0.08133 (9)	0.0193 (4)	
C16	0.4364 (2)	0.39918 (8)	0.20631 (9)	0.0193 (4)	
C17	0.4017 (2)	0.34714 (8)	0.24369 (9)	0.0205 (4)	
C18	0.4090 (3)	0.34667 (8)	0.31982 (9)	0.0227 (4)	
C19	0.4524 (2)	0.39688 (8)	0.36001 (9)	0.0241 (4)	
H19	0.4574	0.3966	0.4121	0.029*	
C20	0.4882 (2)	0.44752 (8)	0.32218 (9)	0.0219 (4)	
C21	0.4809 (2)	0.44985 (8)	0.24629 (9)	0.0208 (4)	
H21	0.5058	0.4854	0.2220	0.025*	
C22	0.3743 (2)	0.37040 (7)	−0.02176 (9)	0.0181 (4)	
C23	0.3613 (2)	0.36860 (7)	−0.10363 (9)	0.0185 (4)	
C24	0.1951 (3)	0.35418 (8)	−0.14347 (9)	0.0219 (4)	
C25	0.1753 (3)	0.35137 (8)	−0.21846 (10)	0.0265 (4)	
H25	0.0583	0.3416	−0.2438	0.032*	
C26	0.3300 (3)	0.36319 (8)	−0.25635 (10)	0.0290 (4)	
H26	0.3189	0.3617	−0.3084	0.035*	
C27	0.5015 (3)	0.37721 (8)	−0.21950 (10)	0.0281 (4)	
H27	0.6082	0.3848	−0.2456	0.034*	
C28	0.5121 (2)	0.37969 (8)	−0.14440 (10)	0.0224 (4)	
H1	0.050 (3)	0.5150 (9)	0.2941 (11)	0.033 (6)*	
H2	0.053 (3)	0.5433 (10)	0.4626 (12)	0.039 (6)*	
H3	0.392 (3)	0.3651 (9)	0.1119 (12)	0.036 (7)*	
H4	0.441 (3)	0.4529 (10)	−0.0251 (12)	0.045 (6)*	

### Atomic displacement parameters (Å^2^)

**Table:**

	*U*^11^	*U*^22^	*U*^33^	*U*^12^	*U*^13^	*U*^23^
Cl1	0.0356 (3)	0.0278 (3)	0.0190 (2)	−0.0018 (2)	0.00479 (18)	−0.00021 (17)
Cl2	0.0346 (3)	0.0393 (3)	0.0171 (2)	0.0015 (2)	0.00166 (18)	−0.00678 (18)
Cl3	0.0359 (3)	0.0224 (3)	0.0360 (3)	−0.0003 (2)	0.0023 (2)	−0.00089 (19)
Cl4	0.0352 (3)	0.0200 (2)	0.0249 (2)	−0.00355 (19)	0.00249 (19)	0.00437 (17)
Cl5	0.0422 (3)	0.0311 (3)	0.0256 (2)	−0.0054 (2)	0.0058 (2)	0.01259 (19)
Cl6	0.0362 (3)	0.0271 (3)	0.0243 (2)	0.0022 (2)	0.00324 (19)	−0.00380 (18)
F1	0.0232 (6)	0.0534 (9)	0.0519 (8)	−0.0052 (6)	0.0122 (5)	−0.0179 (6)
F2	0.0237 (6)	0.0504 (8)	0.0518 (8)	−0.0071 (6)	0.0141 (5)	−0.0213 (6)
F3	0.0211 (6)	0.0405 (7)	0.0298 (6)	−0.0052 (5)	0.0058 (5)	0.0026 (5)
F4	0.0204 (6)	0.0436 (7)	0.0345 (6)	−0.0041 (5)	0.0055 (5)	−0.0001 (5)
O1	0.0437 (8)	0.0258 (7)	0.0185 (6)	−0.0046 (6)	0.0073 (6)	−0.0011 (5)
O2	0.0277 (7)	0.0292 (7)	0.0178 (6)	−0.0046 (6)	0.0037 (5)	−0.0016 (5)
O3	0.0349 (8)	0.0192 (7)	0.0204 (6)	−0.0058 (6)	0.0052 (5)	0.0014 (5)
O4	0.0319 (7)	0.0177 (7)	0.0229 (6)	−0.0010 (5)	0.0050 (5)	0.0041 (5)
N1	0.0256 (9)	0.0223 (9)	0.0166 (7)	−0.0023 (7)	0.0035 (6)	−0.0022 (6)
N2	0.0290 (9)	0.0241 (9)	0.0149 (7)	−0.0038 (7)	0.0048 (6)	−0.0033 (6)
N3	0.0258 (8)	0.0179 (8)	0.0172 (7)	−0.0012 (7)	0.0031 (6)	0.0026 (6)
N4	0.0257 (8)	0.0174 (8)	0.0176 (7)	−0.0017 (6)	0.0039 (6)	0.0034 (6)
C1	0.0236 (10)	0.0244 (10)	0.0184 (8)	−0.0008 (8)	0.0027 (7)	−0.0035 (7)
C2	0.0163 (9)	0.0248 (10)	0.0182 (8)	0.0019 (7)	0.0023 (7)	−0.0053 (7)
C3	0.0180 (9)	0.0248 (10)	0.0212 (8)	0.0025 (7)	0.0032 (7)	−0.0002 (7)
C4	0.0177 (9)	0.0317 (11)	0.0178 (8)	0.0041 (8)	0.0007 (7)	−0.0059 (7)
C5	0.0216 (10)	0.0263 (10)	0.0259 (9)	0.0025 (8)	0.0026 (7)	−0.0067 (7)
C6	0.0179 (9)	0.0216 (10)	0.0286 (9)	0.0031 (7)	0.0031 (7)	−0.0017 (7)
C7	0.0182 (9)	0.0265 (10)	0.0198 (8)	0.0029 (7)	0.0015 (7)	−0.0006 (7)
C8	0.0155 (9)	0.0257 (10)	0.0203 (8)	0.0000 (7)	0.0017 (7)	0.0000 (7)
C9	0.0220 (9)	0.0226 (10)	0.0173 (8)	0.0009 (7)	0.0005 (7)	0.0004 (7)
C10	0.0212 (10)	0.0313 (11)	0.0287 (10)	0.0002 (8)	0.0040 (8)	−0.0021 (8)
C11	0.0374 (12)	0.0305 (12)	0.0325 (11)	0.0085 (9)	0.0101 (9)	−0.0058 (8)
C12	0.0482 (13)	0.0213 (10)	0.0249 (9)	0.0001 (9)	0.0043 (9)	−0.0016 (8)
C13	0.0349 (12)	0.0274 (11)	0.0251 (9)	−0.0068 (9)	0.0028 (8)	0.0001 (8)
C14	0.0235 (10)	0.0278 (11)	0.0250 (9)	−0.0001 (8)	0.0052 (7)	−0.0012 (7)
C15	0.0188 (9)	0.0206 (10)	0.0190 (8)	0.0016 (7)	0.0039 (7)	0.0029 (7)
C16	0.0171 (9)	0.0221 (9)	0.0190 (8)	0.0025 (7)	0.0027 (7)	0.0039 (7)
C17	0.0175 (9)	0.0213 (10)	0.0231 (9)	0.0011 (7)	0.0034 (7)	0.0036 (7)
C18	0.0219 (9)	0.0244 (10)	0.0221 (9)	0.0023 (8)	0.0047 (7)	0.0090 (7)
C19	0.0227 (10)	0.0316 (11)	0.0185 (8)	0.0034 (8)	0.0049 (7)	0.0044 (7)
C20	0.0184 (9)	0.0243 (10)	0.0232 (9)	0.0040 (7)	0.0027 (7)	−0.0003 (7)
C21	0.0199 (9)	0.0206 (9)	0.0223 (9)	0.0028 (7)	0.0039 (7)	0.0047 (7)
C22	0.0156 (9)	0.0177 (9)	0.0214 (8)	0.0036 (7)	0.0032 (7)	−0.0001 (7)
C23	0.0217 (9)	0.0147 (9)	0.0197 (8)	0.0030 (7)	0.0049 (7)	0.0013 (6)
C24	0.0237 (10)	0.0179 (9)	0.0250 (9)	−0.0003 (7)	0.0075 (7)	0.0028 (7)
C25	0.0301 (11)	0.0239 (10)	0.0249 (9)	−0.0028 (8)	−0.0017 (8)	0.0020 (7)
C26	0.0461 (13)	0.0223 (10)	0.0192 (9)	−0.0019 (9)	0.0056 (8)	−0.0004 (7)
C27	0.0328 (11)	0.0251 (11)	0.0283 (10)	−0.0008 (8)	0.0142 (8)	0.0011 (8)
C28	0.0209 (9)	0.0203 (10)	0.0265 (9)	0.0010 (7)	0.0040 (7)	−0.0014 (7)

### Geometric parameters (Å, °)

**Table:**

Cl1—C3	1.7265 (18)	C5—H5	0.9500
Cl2—C4	1.7320 (18)	C6—C7	1.386 (2)
Cl3—C6	1.7317 (19)	C7—H7	0.9500
Cl4—C17	1.7280 (18)	C8—C9	1.493 (2)
Cl5—C18	1.7228 (18)	C9—C10	1.387 (2)
Cl6—C20	1.7362 (19)	C9—C14	1.388 (3)
F1—C10	1.361 (2)	C10—C11	1.372 (3)
F2—C14	1.350 (2)	C11—C12	1.377 (3)
F3—C24	1.357 (2)	C11—H11	0.9500
F4—C28	1.355 (2)	C12—C13	1.383 (3)
O1—C1	1.218 (2)	C12—H12	0.9500
O2—C8	1.224 (2)	C13—C14	1.367 (3)
O3—C15	1.222 (2)	C13—H13	0.9500
O4—C22	1.224 (2)	C16—C21	1.391 (2)
N1—C1	1.355 (2)	C16—C17	1.403 (2)
N1—C2	1.403 (2)	C17—C18	1.389 (2)
N1—H1	0.89 (2)	C18—C19	1.383 (3)
N2—C8	1.360 (2)	C19—C20	1.382 (2)
N2—C1	1.402 (2)	C19—H19	0.9500
N2—H2	0.82 (2)	C20—C21	1.386 (2)
N3—C15	1.354 (2)	C21—H21	0.9500
N3—C16	1.402 (2)	C22—C23	1.493 (2)
N3—H3	0.80 (2)	C23—C24	1.383 (2)
N4—C22	1.360 (2)	C23—C28	1.386 (2)
N4—C15	1.401 (2)	C24—C25	1.369 (2)
N4—H4	0.91 (2)	C25—C26	1.382 (3)
C2—C7	1.387 (2)	C25—H25	0.9500
C2—C3	1.408 (2)	C26—C27	1.389 (3)
C3—C4	1.388 (2)	C26—H26	0.9500
C4—C5	1.377 (3)	C27—C28	1.370 (2)
C5—C6	1.384 (2)	C27—H27	0.9500
			
C1—N1—C2	127.03 (16)	C14—C13—C12	118.06 (19)
C1—N1—H1	113.2 (13)	C14—C13—H13	121.0
C2—N1—H1	119.6 (13)	C12—C13—H13	121.0
C8—N2—C1	128.23 (15)	F2—C14—C13	118.90 (17)
C8—N2—H2	117.9 (15)	F2—C14—C9	117.26 (16)
C1—N2—H2	113.4 (15)	C13—C14—C9	123.81 (18)
C15—N3—C16	128.03 (16)	O3—C15—N3	125.68 (16)
C15—N3—H3	116.0 (16)	O3—C15—N4	119.67 (15)
C16—N3—H3	115.9 (16)	N3—C15—N4	114.65 (15)
C22—N4—C15	128.67 (14)	C21—C16—N3	124.02 (15)
C22—N4—H4	116.5 (14)	C21—C16—C17	119.34 (16)
C15—N4—H4	114.8 (14)	N3—C16—C17	116.64 (15)
O1—C1—N1	125.96 (17)	C18—C17—C16	120.11 (16)
O1—C1—N2	119.17 (15)	C18—C17—Cl4	120.36 (14)
N1—C1—N2	114.85 (15)	C16—C17—Cl4	119.53 (13)
C7—C2—N1	123.82 (15)	C19—C18—C17	120.96 (16)
C7—C2—C3	119.40 (16)	C19—C18—Cl5	119.04 (13)
N1—C2—C3	116.79 (16)	C17—C18—Cl5	120.00 (14)
C4—C3—C2	119.77 (17)	C20—C19—C18	118.02 (16)
C4—C3—Cl1	120.30 (14)	C20—C19—H19	121.0
C2—C3—Cl1	119.93 (14)	C18—C19—H19	121.0
C5—C4—C3	121.06 (16)	C19—C20—C21	122.72 (17)
C5—C4—Cl2	118.68 (14)	C19—C20—Cl6	118.33 (13)
C3—C4—Cl2	120.25 (15)	C21—C20—Cl6	118.94 (14)
C4—C5—C6	118.41 (17)	C20—C21—C16	118.84 (16)
C4—C5—H5	120.8	C20—C21—H21	120.6
C6—C5—H5	120.8	C16—C21—H21	120.6
C5—C6—C7	122.24 (17)	O4—C22—N4	124.46 (16)
C5—C6—Cl3	119.04 (14)	O4—C22—C23	121.38 (15)
C7—C6—Cl3	118.72 (14)	N4—C22—C23	114.16 (14)
C6—C7—C2	119.11 (16)	C24—C23—C28	115.82 (16)
C6—C7—H7	120.4	C24—C23—C22	120.91 (15)
C2—C7—H7	120.4	C28—C23—C22	123.25 (16)
O2—C8—N2	123.81 (17)	F3—C24—C25	118.75 (16)
O2—C8—C9	120.80 (16)	F3—C24—C23	117.71 (15)
N2—C8—C9	115.39 (15)	C25—C24—C23	123.53 (17)
C10—C9—C14	115.37 (17)	C24—C25—C26	118.19 (17)
C10—C9—C8	123.65 (16)	C24—C25—H25	120.9
C14—C9—C8	120.86 (16)	C26—C25—H25	120.9
F1—C10—C11	119.38 (17)	C25—C26—C27	121.07 (17)
F1—C10—C9	117.38 (17)	C25—C26—H26	119.5
C11—C10—C9	123.19 (18)	C27—C26—H26	119.5
C10—C11—C12	118.59 (19)	C28—C27—C26	117.96 (17)
C10—C11—H11	120.7	C28—C27—H27	121.0
C12—C11—H11	120.7	C26—C27—H27	121.0
C11—C12—C13	120.94 (19)	F4—C28—C27	119.34 (16)
C11—C12—H12	119.5	F4—C28—C23	117.21 (15)
C13—C12—H12	119.5	C27—C28—C23	123.43 (17)
			
C2—N1—C1—O1	3.5 (3)	C16—N3—C15—O3	1.6 (3)
C2—N1—C1—N2	−175.52 (16)	C16—N3—C15—N4	−178.88 (16)
C8—N2—C1—O1	175.84 (18)	C22—N4—C15—O3	175.83 (17)
C8—N2—C1—N1	−5.1 (3)	C22—N4—C15—N3	−3.8 (3)
C1—N1—C2—C7	−6.0 (3)	C15—N3—C16—C21	4.5 (3)
C1—N1—C2—C3	173.97 (17)	C15—N3—C16—C17	−176.16 (17)
C7—C2—C3—C4	1.1 (3)	C21—C16—C17—C18	1.0 (3)
N1—C2—C3—C4	−178.90 (16)	N3—C16—C17—C18	−178.33 (16)
C7—C2—C3—Cl1	−178.25 (13)	C21—C16—C17—Cl4	−179.16 (13)
N1—C2—C3—Cl1	1.8 (2)	N3—C16—C17—Cl4	1.5 (2)
C2—C3—C4—C5	−0.9 (3)	C16—C17—C18—C19	−0.8 (3)
Cl1—C3—C4—C5	178.44 (14)	Cl4—C17—C18—C19	179.40 (14)
C2—C3—C4—Cl2	179.03 (13)	C16—C17—C18—Cl5	178.62 (14)
Cl1—C3—C4—Cl2	−1.7 (2)	Cl4—C17—C18—Cl5	−1.2 (2)
C3—C4—C5—C6	0.2 (3)	C17—C18—C19—C20	0.0 (3)
Cl2—C4—C5—C6	−179.71 (13)	Cl5—C18—C19—C20	−179.39 (13)
C4—C5—C6—C7	0.3 (3)	C18—C19—C20—C21	0.5 (3)
C4—C5—C6—Cl3	−179.82 (13)	C18—C19—C20—Cl6	179.69 (14)
C5—C6—C7—C2	−0.1 (3)	C19—C20—C21—C16	−0.3 (3)
Cl3—C6—C7—C2	−179.98 (13)	Cl6—C20—C21—C16	−179.44 (13)
N1—C2—C7—C6	179.38 (16)	N3—C16—C21—C20	178.79 (16)
C3—C2—C7—C6	−0.6 (3)	C17—C16—C21—C20	−0.5 (3)
C1—N2—C8—O2	−2.4 (3)	C15—N4—C22—O4	2.9 (3)
C1—N2—C8—C9	177.94 (17)	C15—N4—C22—C23	−177.49 (16)
O2—C8—C9—C10	121.2 (2)	O4—C22—C23—C24	58.7 (2)
N2—C8—C9—C10	−59.1 (2)	N4—C22—C23—C24	−120.91 (18)
O2—C8—C9—C14	−54.5 (2)	O4—C22—C23—C28	−119.8 (2)
N2—C8—C9—C14	125.14 (18)	N4—C22—C23—C28	60.5 (2)
C14—C9—C10—F1	178.06 (16)	C28—C23—C24—F3	−179.26 (15)
C8—C9—C10—F1	2.1 (3)	C22—C23—C24—F3	2.1 (2)
C14—C9—C10—C11	0.6 (3)	C28—C23—C24—C25	−0.8 (3)
C8—C9—C10—C11	−175.38 (18)	C22—C23—C24—C25	−179.46 (17)
F1—C10—C11—C12	−179.34 (17)	F3—C24—C25—C26	178.90 (16)
C9—C10—C11—C12	−1.9 (3)	C23—C24—C25—C26	0.5 (3)
C10—C11—C12—C13	1.7 (3)	C24—C25—C26—C27	0.4 (3)
C11—C12—C13—C14	−0.1 (3)	C25—C26—C27—C28	−0.8 (3)
C12—C13—C14—F2	−179.42 (17)	C26—C27—C28—F4	178.83 (16)
C12—C13—C14—C9	−1.3 (3)	C26—C27—C28—C23	0.4 (3)
C10—C9—C14—F2	179.24 (16)	C24—C23—C28—F4	−178.09 (15)
C8—C9—C14—F2	−4.7 (3)	C22—C23—C28—F4	0.5 (3)
C10—C9—C14—C13	1.1 (3)	C24—C23—C28—C27	0.4 (3)
C8—C9—C14—C13	177.14 (17)	C22—C23—C28—C27	178.97 (17)

### Hydrogen-bond geometry (Å, °)

**Table:**

*D*—H···*A*	*D*—H	H···*A*	*D*···*A*	*D*—H···*A*
N1—H1···Cl1	0.89 (2)	2.46 (2)	2.9126 (15)	111.8 (16)
N1—H1···O2	0.89 (2)	1.88 (2)	2.641 (2)	141.5 (19)
N2—H2···O1^i^	0.82 (2)	2.00 (2)	2.8205 (19)	173 (2)
N3—H3···Cl4	0.80 (2)	2.43 (2)	2.8944 (16)	118.2 (19)
N3—H3···O4	0.80 (2)	1.99 (2)	2.658 (2)	140 (2)
N4—H4···O3^ii^	0.91 (2)	1.93 (2)	2.8378 (18)	176 (2)

Symmetry codes: (i) −*x*, −*y*+1, −*z*+1; (ii) −*x*+1, −*y*+1, −*z*.
